# Challenges in Providing End‐of‐Life Nursing Care: A Qualitative Content Analysis

**DOI:** 10.1002/nop2.70866

**Published:** 2026-09-30

**Authors:** Zahra Taheri‐Ezbarami, Fateme Jafaraghaee, Ali Karimian Sighlani, Seyed Kazem Mousavi

**Affiliations:** ^1^ Department of Nursing, School of Nursing and Midwifery Guilan University of Medical Sciences Rasht Iran; ^2^ Department of Philosophy University of Guilan Rasht Iran; ^3^ Department of Nursing, Abhar School of Nursing Zanjan University of Medical Sciences Zanjan Iran

**Keywords:** nurses, palliative care, qualitative research, terminal care

## Abstract

**Aim:**

To explore the challenges of providing end‐of‐life nursing care in Iran.

**Design:**

Qualitative content analysis.

**Methods:**

Fifteen participants, including seven nurses, two head nurses, two educational supervisors, two academic staff, one nursing service manager and one critical care physician, were purposively selected from across Iran from October to December 2023. Data were collected through in‐depth semi‐structured interviews in person and by telephone and analyzed using MAXQDA 2020 software using the inductive content analysis approach of Elo and Kingas. We also examined trustworthiness using Elo et al.'s checklist and Lincoln and Guba's criteria.

**Results:**

Four main themes emerged: (1) Emotional burden of caring for dying patients (fear and anxiety, compassion fatigue, moral distress and grief responses); (2) Systemic neglect of end‐of‐life care (educational gaps, lack of national standards and absence of a palliative care organization); (3) Fragmented interdisciplinary care and communication gaps (poor teamwork, physician‐centred system, unclear role definitions and cultural and religious barriers); and (4) Absence of supportive infrastructure (economic, social and technological constraints). Participants believed that these themes were related and mutually reinforcing.

**Conclusion:**

End‐of‐life nursing care in Iran faces serious, interconnected challenges. Therefore, it requires comprehensive institutionalization, planning and educational and clinical interventions.

**Implications for Nursing Practice:**

To improve care quality, nurses need appropriate policies and specific clinical protocols for end‐of‐life care.

**Impact:**

This study examines an important gap in understanding the challenges of end‐of‐life care in Iran and provides evidence for policymaking, education and health system management.

**Reporting Method:**

The study report was guided by the COREQ checklist.

**Patient or Public Contribution:**

No patient or public contribution.

## 1 Introduction

Life and death are complementary concepts in the evolutionary process of all living beings and cannot be considered separately. Even though life evokes enthusiasm and motivation to work in people, death is often seen as a symbol of disintegration and destruction (Fukuda 2023). For this reason, it has been a subject of human concern for centuries and is regarded as a frightening event (Kostka et al. 2021). Today, due to the increase in life expectancy and relative well‐being, more people are treated in different clinical environments in the final stages of their lives, and most deaths occur in conditions where patients are often isolated and under mechanical ventilation. Moreover, their families are concerned about caring for their loved ones (Zhou et al. 2022). During this period, the needs and requests of patients and companions, and the stressfulness of the situation, lead to avoidance of participation in care decisions, dissatisfaction among care workers, neglect of care details, and, as a result, a decrease in the quality of treatment and care (Rumbold and Aoun 2021). Therefore, access to high‐quality, sustainable end‐of‐life care has become an essential concern for policymakers and health managers (Lamppu and Pitkala 2021).

In medicine, palliative care and end‐of‐life care are sometimes used interchangeably, although they differ in several important ways (Gonella et al. 2019). Palliative care can be provided at any stage of chronic or advanced illness, alongside active treatment, and aims to improve quality of life, manage symptoms and provide emotional and psychological support. In contrast, end‐of‐life care is specifically intended for patients approaching the final stage of life, with a greater focus on comfort and easing the dying process (Moazam et al. 2020). A second difference concerns the timing of care. Palliative care may be provided throughout the course of illness, whereas end‐of‐life care generally begins when death is expected in the near future and active treatment is no longer effective (Krau 2016). The two approaches also differ in their focus: palliative care emphasizes symptom management and quality of life, while end‐of‐life care focuses more on relieving distress and supporting patients and families during the final stages of life (Gonella et al. 2022). Although both provide emotional and practical support to families, end‐of‐life care places greater emphasis on helping them cope with the challenges of the dying process. Overall, both types of care aim to provide comprehensive and compassionate support during difficult periods of illness. However, end‐of‐life care is often delivered in the context of complex emotional and ethical challenges, making the provision of optimal care during this transitional stage a significant global challenge (Krau 2016).

According to statistics, about 25 million people worldwide need end‐of‐life care each year (Taheri‐Ezbarami et al. 2024). Despite this, studies show that most health workers, especially nurses, face significant challenges in caring for patients in the final stages of life. These challenges are significantly greater in developing countries (Alqahtani and Mitchell 2019). Recently, national ethical guidelines on supportive and palliative care for patients in the final stages of life have been compiled in Iran (Bagheri et al. 2021). The provision of this type of care has been stated as one of the essential duties of different levels of nursing (Mirzabeigi et al. 2017). Also, according to the Charter of Patient Rights in Iran, receiving care in the final stages of life to maintain comfort is mentioned as one of the fundamental rights of patients (Bagheri et al. 2021).

However, in the few studies conducted, the quality of end‐of‐life care provision in Iran has not been reported to be acceptable (Aghaei et al. 2017; Ghaemizade Shushtari et al. 2022; Ghahramani et al. 2022). A review of these studies reveals several important gaps. First, there is no coherent programme and specific clinical guidelines for the provision of this type of care in Iranian care centres (Ghaemizade Shushtari et al. 2022; Ghahramani et al. 2022). Second, in nursing education, especially in undergraduate curricula, the focus on end‐of‐life care has been very limited (Aghaei et al. 2017; Barasteh et al. 2023). Third, while it is predicted that about one‐third of the Iranian population will reach old age by 2050, the healthcare system does not seem to be ready to meet the growing demand for end‐of‐life services. Fourth, over 98% of Iranians are Muslim and expect to receive end‐of‐life care based on their religious and cultural background (Shiri‐Mohammadabad and Afshani 2021), yet current care practices differ significantly from these expectations.

Although previous studies have examined specific aspects of end‐of‐life care in Iran, including nurses' knowledge (Ghaemizade Shushtari et al. 2022; Damsaz Hafshejani et al. 2026), attitudes (Aghaei et al. 2017), challenges in intensive care units (Akbarian‐Rokni et al. 2023), and the effects of educational interventions (Sohrabi Nasab et al. 2024), a comprehensive and in‐depth analysis of the multifaceted challenges of end‐of‐life care across educational, clinical and policy levels is still lacking. Most existing studies have been quantitative or limited to specific clinical settings, and only a small number of qualitative studies have explored the quality of end‐of‐life care within Iran's healthcare system. Furthermore, the interactions among these challenges—particularly how systemic, organizational and individual factors influence one another and collectively shape the delivery of care—remain poorly understood.

Given the importance of the issue and the identified gaps in the literature, recognizing the potential challenges in providing end‐of‐life care is necessary to improve the quality of care. Qualitative studies, especially content analysis, are the best approach for analyzing events and investigating the visible and hidden dimensions of phenomena (Polit and Beck 2018). Therefore, the researchers decided to plan and conduct the present study to investigate the challenges of providing end‐of‐life nursing care in Iran using this approach.

*Research Question*: This study aimed to address the following research question:

*What are the challenges of providing end‐of‐life nursing care in Iran as perceived by nurses and other healthcare professionals involved in end‐of‐life care?*

## 2 Methods

### 2.1 Study Design

This research is a qualitative content analysis with a conventional approach. Qualitative content analysis is a research method used to discover people's understanding of everyday life phenomena and interpret mental data (Kleinheksel et al. 2020). The complexity of meaning, the holistic view and the dependence of content on context are among the reasons content analysis can be used as a qualitative research method. By doing this, researchers want to understand the behaviour, experiences and concepts used in natural environments (Hosseini et al. 2022). In this method, content collection and analysis are done simultaneously. Content analysis aims to organize and extract meaning from collected information to draw accurate conclusions (Kleinheksel et al. 2020). Therefore, the reason for choosing this approach for the present study was that it allows for the extraction of main themes from the data without imposing preconceived theories, making it particularly suitable for examining less studied phenomena such as the challenges of end‐of‐life care in the Iranian context.

### 2.2 Participants and Sampling

The participants of this research included faculty members, critical care specialists, head nurses, nurses, educational supervisors and nursing service managers from all over Iran who were purposively selected based on the inclusion criteria (having informed consent; having at least 5 years of teaching experience related to the research topic for faculty members; having at least 5 years of clinical work experience in critical care units; having various experiences caring for dying patients for nurses, head nurses, supervisors and nursing service managers; and having a degree in critical care medicine for physicians). Participants were invited by the corresponding author via phone, email and social media. Also, since participants were from all over the country, the research team had no prior professional, hierarchical or supervisory relationships with them.

The first participant was selected based on the study's inclusion criteria and the research team's prior knowledge. He had several years of teaching experience, 18 years as an intensive care unit nurse and educational supervisor, and a strong clinical and educational background in the relevant field. The second participant was selected based on the analysis of the first interview and the research team's knowledge. He was an educational supervisor at one of the educational and medical centres in Rasht, Iran, with more than 20 years of intensive care unit experience and two relatively long years of experience caring for dying relatives. The third participant was a nursing professor with strong knowledge in palliative care. In addition to holding several international certificates in palliative and end‐of‐life care, he had authored numerous books and articles in this field. Subsequent participants were selected based on analysis of previous interviews, consultation with experts and a focus on maximum diversity. Sampling was based on analysis of previous interviews, consultation with experts and a focus on maximum diversity in terms of age, gender, clinical experience, level of education, organizational position and geographic location. Also, one physician was selected from among the participants to capture a multidisciplinary, rather than solely nursing, perspective on the challenges of end‐of‐life care. In addition, four of the participants held international certification in palliative care, and two had experience caring for dying family members within the past year. In addition, all participants had many years of direct clinical experience caring for dying patients and had worked in settings that routinely encountered end‐of‐life situations.

### 2.3 Data Collection

In‐depth, semi‐structured individual interviews were conducted face‐to‐face and over the phone to collect information. The interview guide was developed based on a comprehensive literature review and reviewed for content validity by two qualitative research experts and three palliative care experts. The guide was then pilot tested with two nurses who were not involved in the main study, and minor modifications to the wording and sequence of questions were made before data collection began. Also, in three cases, the researcher contacted them again due to ambiguity in understanding the participants' statements, errors in the information obtained, and the need for further exploration of the issue. It was up to the participants to determine the time and place of the interview, and most face‐to‐face interviews were conducted at their workplace. Before each interview, explanations were provided regarding the research objectives, the reason for recording the interview, voluntary participation, the confidentiality of the information and the interviewees' identities. Permission to record the audio was obtained from them. At the beginning of each interview, participants were asked to introduce themselves briefly, and then questions were asked about demographic characteristics, including age, educational qualification and clinical and educational experience. Then, questions related to the research topic were asked, and the participants' views on the challenges of end‐of‐life care were explored under the interviewer's guidance. The discussion continued until the interviewer was sure they had received the information. The interview form included two categories of open and main questions. Each interview began with this open question: ‘During your years of work and life, have you encountered patients who are passing the last moments of their lives?’ What are your experiences in this field? Then, the main questions included: ‘How do you think the quality of care received by these people is?’ ‘Who do they receive the most care from?’ ‘What are the strengths and weaknesses of this care?’ ‘What are the problems in providing end‐of‐life nursing care?’ The interview times ranged from 30 to 60 min. All interviews were conducted by the corresponding author, who is a qualitative research specialist with 10 years of clinical nursing experience in palliative and end‐of‐life care units, over a 3‐month period from September to November 2023. Also, of the 15 interviews, 9 interviews were conducted in person, and 6 interviews were conducted by telephone, based on participants' preferences and geographic availability. With the participants' permission, interviews were recorded immediately after completion. After listening to them several times, they were transcribed verbatim as soon as possible to provide the necessary feedback for subsequent interviews. Then, data analysis was conducted using MAXQDA 2020 software. Saturation was achieved through ongoing analysis and discussion by the research team, and when two consecutive interviews yielded no new codes or subcategories. However, an additional interview was conducted to ensure saturation and confirm the findings (Rahimi and Khatooni 2024).

### 2.4 Data Analysis

Data analysis was done using Elo and Kyngas's (2008) inductive content analysis approach. According to this method, open coding, coding sheets, grouping, categorization and abstraction are used to develop the model and system or conceptual map of the subject in question. Before starting open coding, the written interview was read several times to find a general sense and immerse in the data. In the open coding stage, initial ideas were extracted from the textual data and listed in a table. After reading each text word by word and line by line several times and pausing enough on the interviews, all the codes related to the research purpose were noted in the margins of the text. Then the interviews were listened to again, and any forgotten points were written down. This process continued continuously from the extraction of the codes to their placement in the respective category. The extracted codes were compared for differences and similarities, and after labelling, they were categorized into subcategories. Then, information grouping was done to reduce the number of categories. More specific labels were used to categorize the data in the next step. In the last stage, that is, abstraction, the researcher achieved a general description of the phenomenon based on the codes and content in the categories and subcategories. It should be noted that all the steps mentioned during the research were carried out several times, back and forth.

### 2.5 Trustworthiness

To ensure data rigour, Elo et al.'s (2014) study checklist was used. This checklist includes three parts: preparation, organization and reporting and has 29 questions. The preparation section includes questions about data collection methods, sampling strategies and the selection of the unit of analysis (Elo et al. 2014). In this research, an attempt was made to increase the rigour of the findings with the help of some strategies, such as using the purposeful sampling method and collecting information from different groups of participants to obtain the most suitable information; establishing proper communication with the participants to create a suitable environment for open and in‐depth interviews; the use of the review method by the participants to confirm the accuracy of the data; the use of semi‐structured questions for better coherence of the interview process; and the clinical and educational background of the researcher regarding the research topic. The second part of the checklist is the organization stage, which includes seven questions regarding the classification and abstraction of findings, their interpretation and representativeness (Elo et al. 2014). In this section, to improve the rigour of the findings, strategies such as participant‐led review to confirm the accuracy of the extracted data and codes, research team review and review by two faculty members with PhD degrees and extensive experience in qualitative research, palliative care and thematic analysis were employed. The third and final part of the checklist is called the report stage, which includes questions about the report's results and analysis (Elo et al. 2014). By using methods such as holding joint meetings of the research team to maximize cooperation and consensus to present the findings in a systematic and orderly manner, trying to report the data clearly and understandably and providing explanations of the process of analyzing the findings in the project report, we tried to increase the rigour of the study in this section.

Additionally, following Lincoln and Guba's (1985) criteria, credibility was established through member checking (12 participants reviewed their transcripts), prolonged engagement (3 months of data collection) and peer debriefing (regular team meetings). For member checking, participants were provided with their full interview transcripts and the preliminary coding summaries. They were asked to confirm the accuracy of both the transcripts and the codes extracted from their responses. Modifications were made based on their feedback. Dependability was ensured through an audit trail and stepwise replication (87% inter‐coder agreement). However, it is important to note that this agreement rate was used as a complementary measure to support consistency, while the primary emphasis was placed on collaborative interpretation and consensus‐building among the research team throughout the coding and categorization process, rather than treating inter‐coder agreement as the sole or primary indicator of rigour. Confirmability was achieved through reflexivity (researcher journals) and respondent validation. Transferability was supported by maximum variation sampling and thick description of the context. Reflexivity was maintained through regular research team meetings where the first author's assumptions, biases and potential influences on data collection and analysis were discussed and documented in a reflexive journal.

### 2.6 Ethical Considerations

This research was approved by the Ethics Committee of Guilan University of Medical Sciences (IR.GUMS.REC.1402.324, available at: https://ethics.research.ac.ir/, Date: September 13, 2023). Informed consent was obtained from all participants before conducting the interviews. Written consent was obtained for recording face‐to‐face interviews, and verbal consent with permission to record was obtained for telephone interviews. This process involved all institutions participating in the study.

## 3 Results

In this study, 15 participants were included: seven nurses, two head nurses, two nursing educational supervisors, two nursing faculty members, one nursing service manager and one critical care physician, aged 33–60 years. The participants had 10–29 years of work experience. They also worked at Guilan, Dezful, Baqiyatullah, Birjand, Zanjan and Ahvaz Universities of Medical Sciences. Table 1 provides more information on the participants' demographic characteristics.

**TABLE 1:** Demographic characteristics of participants.

Participant code	Age (years)	Gender	Marital status	Education level	Organizational position	Experience as a faculty member	Clinical experience
1	49	Female	Married	PhD	Faculty member	11	18
2	53	Female	Married	MSN	Nursing educational supervisor	—	29
3	51	Female	Married	MSN	Nurse	—	26
4	60	Female	Married	PhD	Faculty member	21	8
5	48	Female	Single	BSN	Nurse	—	24
6	38	Female	Married	BSN	Head nurses	—	15
7	49	Female	Married	BSN	Nurse	—	26
8	60	Female	Married	MSN	Head nurses	—	22
9	33	Female	Single	BSN	Nurse	—	10
10	34	Male	Married	MSN	Nurse	—	12
11	39	Female	Married	BSN	Nurse	—	15
12	46	Female	Married	BSN	Nurse	—	23
13	53	Male	Married	Specialist	Critical care physician	12	7
14	52	Female	Married	MSc	Nursing service manager	—	28
15	47	Female	Married	MSc	Nursing educational supervisor	—	26

At the beginning of the coding process, 74 primary codes with a frequency of 717 cases were extracted from the interview transcripts. Following several rounds of revision—including expert consultation, reduction, deletion and merging of overlapping codes—the final dataset comprised 61 codes with a frequency of 663 cases. These codes were then organized into 25 subcategories, 8 categories and 4 main themes: (1) emotional burden of caring for dying patients, (2) systemic neglect of end‐of‐life care, (3) fragmented interdisciplinary care and communication gaps and (4) absence of supportive infrastructure (Table 2). These themes reflect the multilayered challenges that nurses and other healthcare professionals encounter when providing end‐of‐life care within the Iranian context.

**TABLE 2:** Codes, subcategories, categories and themes regarding the challenges of end‐of‐life nursing care in Iran.

Theme	Category	Subcategory	Some codes
Emotional burden of caring for dying patients	Intrapersonal	Fear and anxiety	Fear of providing end‐of‐life care, performance anxiety, helplessness in confronting death
		Compassion fatigue	Gradual erosion of empathy, emotional exhaustion, feeling overwhelmed by repeated exposure to suffering
		Moral distress	Ethical conflict from providing futile treatments, values violation, powerlessness in decision‐making
		Grief responses	Unresolved grief from patient deaths, suppressed emotions, accumulation of sorrow over time
Systemic neglect of end‐of‐life care	Education	Curricular deficiencies	Lack of relevant training during education and work, insufficient attention in the curriculum, lack of regular refresher courses, more focus on postgraduate courses instead of bachelor's degrees
		Faculty limitations	Weak knowledge of faculty members in teaching relevant courses, weak current teaching methods, gap between theory and practice
	Treatment	Absence of national standards	Lack of adequate rules, guidelines and standards; lack of clinical guidelines
		No palliative care organization	Lack of a separate organization for palliative care and end‐of‐life at the national level
		Specialist shortage	Lack of specialist nurses in palliative care and end‐of‐life
Fragmented interdisciplinary care and communication gaps	Care team fragmentation	Poor professional communication	Weak professional communication, lack of clarity in job descriptions, physician‐centred treatment system
		Unclear role definitions	Ambiguity in responsibilities, confusion about who should talk to families, delegation confusion
		High workload and stress	High workload during shifts, stressful work environment, insufficient monitoring of nurses' performance
		Weak teamwork	Lack of interdisciplinary collaboration, inappropriate role models among hospital nurses
	Communication gaps with patients and families	Cultural and religious barriers	Cultural differences, religious differences between nurse and patient, cultural taboos around death, religious misconceptions
		Physical and mental limitations	Physical and mental conditions of the patient (reduced consciousness, cognitive impairment), inability to express needs
		Inadequate communication skills	Lack of proper communication, inadequate understanding of the patient's needs and requests, lack of attention to the patient's human dignity
		Overly involved families	Excessive involvement of companions, unrealistic expectations of families, demands on nurse's time
		Family conflicts	Disagreements among family members about treatment decisions, lack of surrogate decision‐maker, cultural expectations
		Lack of family support protocols	No training on how to communicate with families, no structured family meetings, lack of bereavement support
Absence of supportive infrastructure	Social	Gradual changes	Increasing number of chronic diseases, general movement of population towards old age, increase in patients needing end‐of‐life care
		Sudden crises	Negative effects of COVID‐19 pandemic
	Economic	Macroeconomic problems	Governments' lack of attention due to unfavourable economic conditions, incomplete health insurance coverage, sanctions effects
		Microeconomic problems	Unfavourable economic conditions for nurses, low salaries, economic problems of families
	Infrastructure	Hardware issues	Low development of treatment departments, lack of palliative care and end‐of‐life departments in hospitals, no hospices, lack of home care development
		Software issues	Weakness in using new technologies in providing these care services, poor telehealth, no home visiting programmes

### 3.1 Theme 1: The Emotional Burden of Caring for Dying Patients

The first theme was the narrative of nurses enduring a profound psychological burden of caring for dying patients. Participants described intense fear, anxiety and helplessness when faced with death. This burden was not limited to the initial experiences but continued to accumulate over time. In the Iranian context, ‘emotional burden’ emerged as a multidimensional concept that encompassed not only immediate emotional reactions to a patient's death but also the cumulative psychological stress resulting from repeated exposure to suffering, cultural expectations and beliefs about death and dying and the moral tensions resulting from providing care in a system with limited resources. Participants explained that this emotional burden manifested itself in four interrelated subcategories: fear and anxiety, compassion fatigue, moral distress and grief reactions.

One participant stated:

Every death you see changes you. After 20 years, I am not the same person I was when I started this job – I have become more cautious, but of course I have to admit I am still scared despite having seen so much. (Participant 2, Educational Supervisor, 29 years of experience)

The subcategory of ‘fear and anxiety’ was not limited to the fear of death, but also included anxieties related to professional performance, including fear of making mistakes, concerns about not knowing how to talk to families about the patient's condition and fears of being unable to provide adequate comfort and care. As one participant explained:

I remember my first patient who died. I was 23 years old. He asked me, ‘Am I dying?’ I was stunned. I didn't know what to say. I didn't have the knowledge to respond to him honestly but compassionately. I just stood there until another nurse came. That moment of helplessness still haunts me – not because I couldn't save him, but because I couldn't empathize with him. And that's what we're supposed to do, right? Be there? (Participant 9, nurse, 10 years' experience)

‘Compassion fatigue’ emerged as a gradual erosion of empathic capacity due to repeated exposure to patients' suffering. Participants spoke of the emotional exhaustion and psychological strain of constantly caring for dying patients, especially over long years of service. One participant stated:

After 26 years, I feel like I have nothing left to offer. I used to cry with families, but now I just do my job out of compulsion. It's not that I don't care; I care a lot, but I've learned to protect. myself by numbing myself.” (Participant 7, nurse, 26 years of experience)

‘Moral distress’ was another important subcategory that emerged when nurses were forced to provide treatments that they considered useless or inconsistent with their professional values. This is particularly important in the Iranian context, where cultural and religious values emphasize the sanctity of life. At the same time, in the absence of clear ethical guidelines on limiting or terminating care, continuing invasive life‐sustaining interventions can place significant moral strain on healthcare professionals.

We keep patients alive for weeks, sometimes months, on life support, when everyone knows there is no hope. Families want everything done, because they believe God will save them. Doctors don't want to be the ones to say, ‘Enough is enough’. And I'm the one who has to. see this suffering and pretend that this is care. It's exhausting; not physically, but morally.” (Participant 12, nurse, 23 years of experience)

‘Grief responses’ formed the fourth subcategory, which included unresolved grief over patient deaths, repressed emotions and the gradual accumulation of grief over time. Participants spoke of repeatedly being confronted with patient deaths without adequate time to process these experiences or receive emotional support.

I've seen hundreds of patients die; hundreds. And no one has ever asked me how I feel about it. We're expected to move on to the next patient and the next shift, as if nothing had. happened. But those deaths stay with you; they build up inside you.” (Participant 5, nurse, 24 years of experience)

Indeed, emotional stress was compounded by inadequate knowledge and skills, creating a cycle in which lack of competence exacerbated fear, and fear, in turn, prevented learning. Subcategories of this theme included: fear and anxiety, compassion fatigue, moral distress and grief reactions. Participants described how the cumulative weight of these emotional experiences leads to emotional exhaustion and, in some cases, even a desire to leave the profession.

### 3.2 Theme 2: Systematic Neglect of End‐of‐Life Care

Participants felt that there was a structural weakness in the healthcare system's prioritization of end‐of‐life care. In the education sector, participants believed that death and dying were largely ignored in the curriculum:

In four years of nursing school, we had maybe one lecture on terminal care. It's like it doesn't exist. We learn everything about saving lives, but nothing about supporting people through death. (Participant 9, Nurse, 10 years' experience)

Faculty members noted that even when they recognized the importance of this topic, the absence of national curriculum requirements prevented its integration:

I would like to teach more about death and dying, but by law I have to follow the official curriculum of the country. The Ministry of Health curriculum also covers very little about this topic. Even though I have been teaching for 21 years, I have never taught several separate sessions on palliative care. (Participant 4, faculty member, 21 years of teaching experience)

In clinical settings, participants also noted a lack of a national palliative care organization, standard clinical guidelines and specialist nurses. One participant stated:

In this system, end‐of‐life care is the second‐highest priority. We have to accept that every patient will die one day. Why do we only have life plans? Why are we not serious about end‐of‐life? Why don't we have good educational content, guidelines, and trained professionals in this area? (Participant 4, faculty member, 21 years of teaching experience)

This systemic neglect was described as institutional indifference—a failure to recognize end‐of‐life care as a legitimate priority worthy of policy attention and resource allocation. Subcategories within this theme included: Curricular Deficiencies, Faculty Limitations, Absence of National Standards and No Palliative Care Organization.

### 3.3 Theme 3: Fragmented Interdisciplinary Care and Communication Gaps

This theme reflected two interrelated dimensions of communication breakdowns in end‐of‐life care. The first dimension addressed fragmentation within the interdisciplinary care team, characterized by weak professional communication, unclear role definitions, a physician‐centred system and high workload. The second dimension addressed communication gaps with patients and families, characterized by cultural and religious barriers, inadequate communication skills, patient‐related limitations and family‐related challenges. These two dimensions are closely interconnected: when the care team does not communicate effectively among themselves, they cannot communicate effectively with patients and families.

### 3.4 Fragmentation Within the Care Team

Participants stated that in Iran, professional communication is weak, role definitions are unclear, and the healthcare system is physician‐centred. In such a situation, nurses are systematically marginalized:

Doctors come and give orders and go. Nurses are expected to follow without question. There is no real teamwork. I have been in this field for 29 years, and it has gotten worse every year. (Participant 2, Head Nurse)

Another participant said:

We don't really know who is supposed to talk to the patient's family about death. Doctors say it's the nurse's job; nurses say the doctor is directly responsible for the patient's condition. So sometimes no one explains the patient's condition to the caregivers, and sometimes the doctor or the nurse tells them different things. (Participant 8, head nurse, 22 years of experience)

This fragmentation of roles is exacerbated by heavy workloads and stressful work environments, which also saps the energy left for teamwork:

When there is no team communication, the patient sees the real damage. We all repeat things or don't do things. Sometimes our opinions differ. The problem is that we really want to work together, but our work system doesn't give us the time or structure to do it properly. (Participant 12, nurse, 23 years of experience)

Among other things, the physician‐centred nature of the healthcare system was identified as a key factor in this fragmentation:

As a nurse, you have no role in the patient's treatment planning. You are expected to just follow the doctor's orders. Even when you disagree. Your opinion doesn't matter. Even the patient feels that. They see that nurses have no real power. (Participant 12, nurse)

### 3.5 Communication Gaps With Patients and Families

Participants explained how cultural taboos around death make it very difficult to have open and honest conversations:

In Iranian culture, death is not talked about much. Even if you do talk about it, sometimes the families get very upset. They say, ‘Don't talk about it, it's not good for you’. That's why many nurses avoid talking about it altogether. Of course, this avoidance does not make it unimportant. The patient is dying and we are very upset with him. We just pretend that nothing happened. (Participant 7, nurse, 26 years of experience)

Participants also mentioned poor communication skills and a lack of training in delivering bad news or responding to families' emotional needs. They believed that the physical and psychological conditions of dying patients, such as decreased consciousness and cognitive impairment, make these communications more difficult. Meanwhile, families also clashed over issues, which put nurses in a difficult position to mediate:

The family asks me, ‘Is my mother in pain?’ To be honest, I have no training on how to answer this question correctly. If I say yes, they get upset. If I say no, I'm lying. I've learned not to talk. So, they get upset with me. (Participant 3, nurse, 26 years of experience)

Religious and belief differences between nurses and patients also posed some barriers:

When a patient's religious beliefs are different from mine, I don't know how to support them spiritually. I want to help them, but I'm afraid of saying the wrong thing or hurting them. (Participant 10, nurse, 12 years of experience)

Subcategories of this theme included: poor professional communication, unclear role definitions, physician‐centred system, high workload and stress, cultural and religious barriers, inadequate communication skills, patient‐related barriers (decreased alertness and cognitive impairment) and family‐related barriers (overly involved families, family conflicts and unrealistic expectations).

### 3.6 Theme 4: Lack of Supportive Infrastructure

This theme refers to the lack of macro‐level resources that make end‐of‐life care difficult. Participants identified three dimensions: social, economic and infrastructural.

*Social challenges*: The ageing population, coupled with the rise in chronic diseases, has increased the demand for end‐of‐life care, but the healthcare system is not responding:

On the one hand, the pattern of diseases in our society has changed—autoimmune diseases, cancers, chronic diseases. On the other hand, the number of patients with these conditions has increased. Maybe one reason is that our society is aging. Before, when we were working, we didn't have so many sick patients. Now, every shift, every day, we're caring for patients who are dying. (Participant 14, Nursing Service Manager)

*Economic constraints*: International sanctions, budget deficits and lack of full insurance coverage have limited investment in palliative and end‐of‐life care:

With the current economic situation, there is no priority for palliative care. Even basic equipment is barely available. It must be said that there are so many problems in the Iranian health system that no macro‐level manager gives them any importance. We are now struggling with our core tasks. Providing palliative and end‐of‐life care primarily requires money; it requires budgets, and we also have severe constraints in this regard. (Participant 13, Critical Care Physician)

*Infrastructure deficits*: The absence of hospices, limited home care services and weak technology infrastructure force dying patients into acute care settings where end‐of‐life care is not prioritized:

We don't have the proper infrastructure to start a palliative care unit. That's why we admit the dying patient among other patients. Since we don't have hospice at all. We don't have good care at home either. So the ICU becomes the default place for dying—and we're not set up for that. (Participant 5, Nurse, 24 years' experience)

Subcategories of this theme included social challenges (ageing population and chronic diseases), economic constraints (sanctions, budget deficits and incomplete insurance) and infrastructure deficiencies (lack of nursing homes, limited home care and poor technology).

### 3.7 Interrelationships Among Themes

The research team's interpretive analysis revealed perceived interconnections among the four themes. Rather than operating in isolation, the challenges participants described appeared to form a complex, mutually reinforcing pattern (Figure 1). However, these relationships reflect participants' perceptions and the research team's interpretation of their accounts, not demonstrated causal pathways.

**FIGURE 1:**
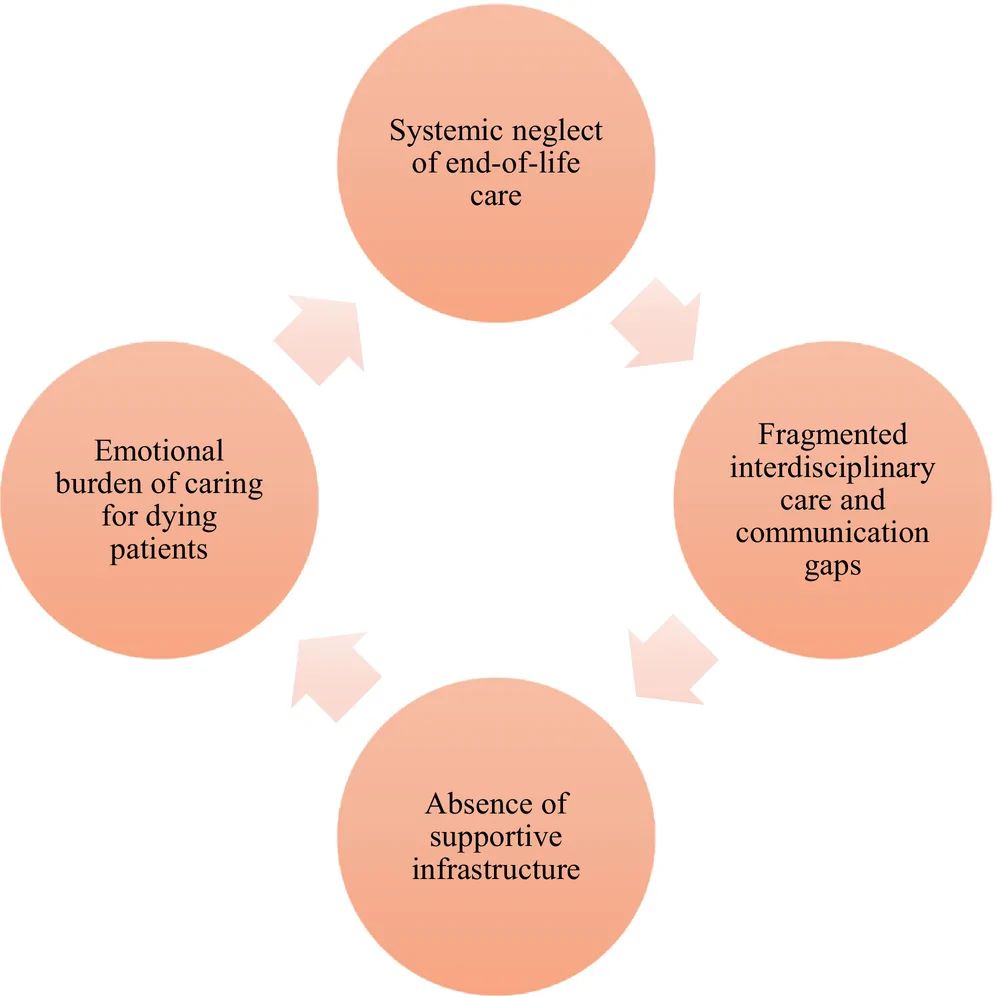
Conceptual model of the interrelationships between the main challenges in providing end‐of‐life care in Iran, according to participants' perspectives. Arrows represent perceived connections described by participants, not verified causal pathways.

First, several participants described how emotional burden and communication difficulties contributed to their intention to leave their units. They felt that this, in turn, could lead to staff shortages and exacerbate existing staffing problems. However, the present qualitative design does not permit causal inference, and these relationships should be interpreted as participants' perceptions rather than demonstrated causal pathways. One participant explained:

Due to economic problems in Iran, faculty members leave the university with knowledge. Therefore, we are faced with new graduates who are weaker year by year. Of course, they also leave the organization after a few years of acquiring knowledge and skills. Therefore, the number of experts is really limited. (Participant 12, nurse, 23 years of experience)

Second, participants described how systemic neglect appeared to contribute to a heavy emotional burden on nurses. They explained that the lack of formal education about end‐of‐life care left them feeling unprepared, which they felt exacerbated fear, anxiety and low self‐confidence. One participant described this experience:

After seeing my first patient die, I never wanted to see another one die. To be honest, it wasn't because I was lazy. It was because I was afraid. That fear kept me from learning about it. That fear still haunts me, and it has kept me from knowing much about this care. (Participant 5, nurse, 24 years of experience)

Third, fragmented interdisciplinary care and communication gaps appeared to serve as a channel through which structural and organizational problems became visible at the clinical level. Participants perceived that poor communication and teamwork contributed to ambiguity in responsibilities and physician‐centred decision‐making. According to their accounts, these factors, in turn, created gaps in communication with patients and families. Cultural and religious barriers, inadequate communication skills and family‐related challenges further complicated these communication gaps.

Fourth, participants perceived a relationship between the absence of supportive infrastructure—particularly economic constraints and infrastructure deficiencies—and insufficient attention to end‐of‐life care. According to their accounts, without dedicated palliative care organizations or standard guidelines, training programmes remain scattered, and healthcare teams lack clear protocols for providing care.

Finally, participants perceived that organizational challenges—such as systemic neglect and fragmented care—may reduce momentum for structural investment. According to their accounts, this creates what they described as a self‐perpetuating pattern: structural and organizational challenges appear to contribute to personal difficulties for nurses, which they felt, in turn, worsened organizational problems by increasing burnout and turnover. While similar patterns have been reported elsewhere (Nelson et al. 2010), the present study offers an interpretive representation of these perceived relationships rather than a demonstrated causal model (Figure 1).

## 4 Discussion

This study examined the main challenges of providing end‐of‐life nursing care in Iran. It found four key issues: Emotional burden of caring for dying patients, systemic neglect of end‐of‐life care, fragmented interdisciplinary care and communication gaps and absence of supportive infrastructure.

The emotional burden of caring for dying patients is a persistent challenge in studies. Kostka et al. (2021) found that nurses often experience fear, anxiety and helplessness when dealing with dying patients. Royani et al. (2024) noted that repeated exposure to patients' deaths is a difficult and daunting experience for nurses and can reduce their motivation and self‐confidence and sometimes even lead to emotional distancing between the nurse and the patient and family as a defence mechanism. Despite this, our study presented a new finding: emotional stress in Iranian nurses was two‐way. It stemmed from both the direct experience of caring for the patient. Systemic factors, such as poor academic training, lack of organizational support and cultural expectations around death, also contributed. As a result, this stress was widespread and multilayered.

Moral distress is defined as a situation in which an individual knows what is morally right to do but cannot do so because of constraints and obstacles (Jameton 1984; Epstein and Hamric 2009). This concept emerged as an important part of the emotional burden in Iranian nurses. Participants stated that they were sometimes forced to provide invasive and unpleasant treatments that they considered futile, but were forced to continue them by family members because these families considered the cessation of treatment and care to be contrary to their religious beliefs. In fact, this conflict between professional principles and family demands creates great psychological distress. Contrary to findings in some Western societies, where moral distress is often linked to resource constraints, in the Iranian context this distress was closely related to religious and cultural values and their influence on treatment expectations. In addition to moral distress, participants described fear and anxiety related to feelings of inadequacy in professional performance, compassion fatigue from repeated exposure to patients' suffering and unresolved grief from repeated patient deaths; experiences that were exacerbated by the lack of adequate emotional support and opportunity to process these experiences. The cumulative nature of these emotional challenges suggests that interventions should not focus solely on individual coping strategies but should also include organizational mechanisms to support staff emotionally. These mechanisms could include regular debriefing and processing sessions, access to psychological counselling and structured ethical guidance for end‐of‐life care decision‐making.

This study found systematic neglect at different levels of the Iranian healthcare system. In the education sector, participants felt that basic nursing curricula did not adequately cover end‐of‐life care, especially at the undergraduate level. Similarly, Barasteh et al. (2023), in a review study, noted minimal training in palliative and end‐of‐life care in Iranian nursing programmes. Participants also stated that they have long understood this necessity but should be taught according to the existing curriculum requirements, which prioritize acute care content over technical and end‐of‐life care. The consequence of this educational gap is that nurses enter the clinical environment without the necessary knowledge and skills to provide competent end‐of‐life care, which in turn exacerbates fear, anxiety and moral distress.

At the clinical level, the study noted a lack of national protocols, clinical guidelines and dedicated organizational facilities for palliative and end‐of‐life care. Unlike countries such as the United Kingdom or Australia, participants perceived that Iran lacks a national systematic structure for palliative and end‐of‐life care. In line with this finding, Bagheri et al. (2021) stated that despite national ethical guidelines, palliative care in Iran remains limited and under‐resourced. Furthermore, the lack of an independent national organization for palliative and end‐of‐life care means no centralized entity oversees policy development, resource allocation, workforce planning or quality improvement. The lack of attention to this care in Iran is deeply concerning. This country has one of the highest rates of population ageing in the world.

Furthermore, the predominantly Muslim population and the specific cultural context of the country require a local programme in this field. Therefore, this gap between the realities and existing policies indicates that end‐of‐life care is not yet considered a healthcare priority in Iran. Therefore, multifaceted policy interventions should address this issue, including establishing an independent national organization for palliative and end‐of‐life care, integrating relevant educational content into nursing curricula at all levels and developing national standards and clinical guidelines based on available evidence.

Fragmented Interdisciplinary Care and Communication Gaps refers to two closely related aspects of communication disorders: (1) the fragmentation that occurs within interdisciplinary care teams and (2) the communication gaps that exist with patients and families. Although these two aspects are described separately, they are closely linked because poor communication and coordination within the team can directly affect the team's ability to communicate effectively with patients and families.

Fragmentation within interdisciplinary care teams was evident in the way professionals communicated and worked together. Participants described unclear roles, overlapping responsibilities and a tendency for decisions to remain largely physician‐led. Heavy workloads appeared to add to these difficulties. Several participants felt that, despite being closely involved in patient care, nurses did not always have their views taken seriously in clinical decision‐making. This hierarchical structure, in which physicians occupy the top position and nurses often feel marginalized, reflects deeper structural issues in how healthcare is organized in Iran. The physician‐centred approach means that nurses' input is often ignored, even when nurses have more direct and continuous contact with dying patients and their families. This hierarchical structure also creates communication gaps: when physicians do not communicate effectively with nurses, nurses cannot communicate effectively with patients and families. Akbarian‐Rokni et al. (2023) reported similar concerns in intensive care settings, and studies from other countries have also noted limited recognition of nurses' contributions in physician‐centred healthcare systems (Allen 2014; Harris 2020).

However, this study's findings point to an additional concern in end‐of‐life care. At this stage, decisions are often ethically and clinically complex and require team members to communicate and work closely together. When responsibilities are not clearly assigned, uncertainty can also arise about who should speak with families about the patient's prognosis or available treatment options. In these situations, nurses may end up managing these difficult conversations on their own, often without sufficient preparation, guidance or organizational support.

Communication gaps with patients and families mostly stemmed from cultural and religious barriers. Participants felt that talking about death and dying is sensitive in Iranian culture. They stated that in many situations, talking openly about death is considered inappropriate, and some families believe that discussing such a topic is ominous and reduces the patient's and family's hopes and resistance. They even sometimes ask nurses to give false hope and lie to the patient instead of honestly stating the patient's prognosis. Similar findings have been reported in other Middle Eastern countries (Khonsari et al. 2024; Ekberg et al. 2021). However, in the present study, participants explained how Iran's cultural and religious context, especially the Shiite context, influences the formation and occurrence of these issues. Belief in divine will and the sanctity of life were often closely related to families' desire to protect the patient from distressing and difficult information. This dichotomy placed nurses in a difficult position, as they were conflicted between families' requests for confidentiality and their professional responsibility to provide honest information. Participants also noted a lack of adequate training in culturally sensitive care. They believed that nurses received no training on how to deliver bad news, how families might respond emotionally, and the role of religious beliefs in end‐of‐life care.

Absence of supportive infrastructure is another major challenge, as seen in other developing countries. Alqahtani and Mitchell (2019) found that limited resources make end‐of‐life care harder in these places. Ghanbari et al. (2017) showed that although healthcare costs in Iran have increased, funding has not kept pace. This study found that without hospices, with little home care, and not enough technology, dying patients often end up in hospitals that are not set up for palliative care. This is called the “acute care bias” (Kelley and Morrison 2015), and it is especially strong in Iran, where there are no hospices and home care is rare. These gaps worsen other problems: nurses are overworked, patients feel isolated, families are stressed and care quality drops. Fixing these problems will need investment in palliative care centres, home care and technology. But due to economic sanctions and budget constraints, creative solutions such as integrating palliative care into primary care may be needed.

This study found that, from the participants' perspective, the four main challenges of end‐of‐life nursing care are interconnected and mutually reinforcing (Figure 1). They believed that poor infrastructure can increase organizational problems and pressure on nurses, leading to burnout and staff turnover, which can, in turn, exacerbate existing problems. Although previous studies have suggested this “vicious cycle” (Nelson et al. 2010), it has not been reported in Iran. Therefore, improving end‐of‐life care requires simultaneous attention to educational, organizational and structural factors (Ferlie and Shortell 2001).

This study is the first to map out these challenges in Iran using qualitative analysis. Earlier research mostly relied on surveys or focused on specific settings, such as intensive care units (Ghaemizade Shushtari et al. 2022; Ghahramani et al. 2022). This study examines challenges in education, care and infrastructure at the national, organizational and personal levels. It also highlights emotional strain as a key issue, including fear, anxiety, compassion fatigue, grief and moral distress and shows how these affect nurses. The study also points out communication barriers unique to Iran, such as cultural and religious barriers to open communication about death, which have not been reported before. Finally, it offers a model showing how these challenges interact and provides a framework for developing solutions at different levels.

Although recent qualitative studies in Iran have examined end‐of‐life care by focusing on patients (Akbarian‐Rokni et al. 2023) or nurses (Royani et al. 2024), this study differs in several respects. First, by examining different aspects of life‐threatening care simultaneously, it provides a more comprehensive picture of the challenges of this care. Second, the geographical and professional diversity of the participants provides a more comprehensive perspective than studies limited to a single centre. Third, this study highlights the relationship between challenges and the role of cultural and religious factors in the difficulty of discussing death, which have received less attention in previous studies in Iran. Finally, the interview data presented here have not been published or presented elsewhere.

### 4.1 Limitations

This study has several limitations. Given the qualitative nature of the research, the findings are context‐specific and may not generalize to all settings; however, a detailed description of the data and the diversity of participants may increase their transferability to similar settings. Also, despite participation from different regions, the relatively centralized structure of the Iranian health system may mean some of these challenges are similar in other regions. On the other hand, the study focused mainly on nurses' perspectives and did not include those of patients and families. The presence of only one critical care physician may also have limited the diversity of medical perspectives. Therefore, future studies should include broader participation from physicians, patients, families and other members of the treatment team to provide a more comprehensive picture of end‐of‐life care challenges.

### 4.2 Implications for Nursing Practice

This study's findings indicate that improving end‐of‐life nursing care requires attention to several areas. Proposed solutions include establishing a national institution to plan and monitor palliative and end‐of‐life care, incorporating these topics into nursing education programmes, holding training courses for medical staff, developing guidelines appropriate to the country's cultural and religious conditions and expanding services such as hospice and home care. Addressing these issues can improve care quality and provide better conditions for end‐of‐life patients. However, more studies are necessary to examine the feasibility and effectiveness of these solutions in the Iranian health system.

## 5 Conclusion

This study found serious challenges in providing end‐of‐life care in Iran, categorized into four interconnected themes: the emotional burden of caring for dying patients, systemic neglect of end‐of‐life care, fragmented interdisciplinary care and communication gaps and a lack of supportive infrastructure. Participants described these contexts as interrelated and mutually reinforcing. Therefore, comprehensive, coordinated solutions at all levels can be more effective than separate, isolated interventions. However, these strategies require further evaluation in Iran. This study's findings can help policymakers, planners and micro‐ and macro‐level managers in the education and health sectors take more effective measures to reduce and address these challenges. Undoubtedly, improving the quality of this type of care can reduce the suffering of patients and their families and improve the standards of nursing care in Iran.

## Autho.r Contributions

**Zahra Taheri‐Ezbarami:** conceptualization, writing – original draft, methodology, software, data curation. **Fateme Jafaraghaee:** conceptualization, writing – original draft, methodology, writing – review and editing, data curation. **Ali Karimian Sighlani:** conceptualization, methodology, writing – review and editing, data curation. **Seyed Kazem Mousavi:** conceptualization, methodology, investigation, funding acquisition, writing – original draft, software, data curation, supervision.

## Funding

The authors have nothing to report.

## Disclosure

No generative artificial intelligence was used in the writing or analysis of this manuscript.

## Ethics Statement

This study was approved by the Research Department and Ethics Committee of Guilan University of Medical Sciences (Approval No. IR.GUMS.REC.1402.324, Date: September 13, 2023, available at: https://ethics.research.ac.ir/). The approval covered recruitment and data collection across all participating institutions. The study was conducted in accordance with the Declaration of Helsinki.

## Consent

All participants received detailed oral and written information about the study objectives, the voluntary nature of participation, their right to withdraw at any time without consequences, and the confidentiality of their data. Written informed consent was obtained from all participants prior to the interviews. Participants were also asked for separate permission to audio‐record the interviews; written consent was obtained from participants who were interviewed face‐to‐face, and verbal consent was obtained and audio‐recorded for telephone interviews. All identifying information was removed from transcripts, and participants were assigned code numbers.

## Conflicts of Interest

The authors declare no conflicts of interest.

## Data Availability

The data that support the findings of this study are available from the corresponding author upon reasonable request.
